# Identification of a Novel PPAR Signature for Predicting Prognosis, Immune Microenvironment, and Chemotherapy Response in Bladder Cancer

**DOI:** 10.1155/2021/7056506

**Published:** 2021-12-30

**Authors:** Ke Zhu, Wen Deng, Hui Deng, Xiaoqiang Liu, Gongxian Wang, Bin Fu

**Affiliations:** ^1^Department of Urology, The First Affiliated Hospital of Nanchang University, China; ^2^Jiangxi Institute of Urology, Nanchang, Jiangxi 330006, China

## Abstract

**Background:**

Mounting evidence has confirmed that peroxisome proliferator-activated receptors (PPARs) played a crucial role in the development and progression of bladder cancer (BLCA). The purpose of this study is to comprehensively investigate the function and prognostic value of PPAR-targeted genes in BLCA.

**Methods:**

The RNA sequencing data and clinical information of BLCA patients were acquired from The Cancer Genome Atlas (TCGA). The differentially expressed PPAR-targeted genes were investigated. Cox analysis and least absolute shrinkage and selection operator (LASSO) analysis were performed for screening prognostic PPAR-targeted genes and constructing the prognostic PPAR signature and then validated by GSE13507 cohort and GSE32894 cohort. A nomogram was constructed to predict the outcomes of BLCA patients in combination with PPAR signature and clinical factors. Gene set enrichment analysis (GSEA) and immune cell infiltration were implemented to explore the molecular characteristics of the signature. The Genomics of Drug Sensitivity in Cancer (GDSC) database was used to predict the chemotherapy responses of the prognostic signature. The candidate small molecule drugs targeting PPAR-targeted genes were screened by the CMAP database.

**Results:**

We constructed and validated the prognostic signature comprising of 4 PPAR-targeted genes (CPT1B, CALR, AHNAK, and FADS2), which was an independent prognostic biomarker in BLCA patients. A nomogram based on the signature and clinical factors was established in the TCGA set, and the calibration plots displayed the excellent predictive capacity. GSEA analysis indicated that PPAR signature was implicated in multiple oncogenic signaling pathways and correlated with tumor immune cell infiltration. Patients in the high-risk groups showed greater sensitivity to chemotherapy than those in the low-risk groups. Moreover, 11 candidate small molecule drugs were identified for the treatment of BLCA.

**Conclusion:**

We constructed and validated a novel PPAR signature, which showed the excellent performance in predicting prognosis and chemotherapy sensitivity of BLCA patients.

## 1. Introduction

Bladder cancer (BLCA) is one of the common causes of cancer-related deaths with elevated heterogeneity, accounting for over 200,000 cancer-related deaths in 2020 [1]. According to the tumor with or without muscle invasion, BLCA is classified into non-muscle-invasive BLCA (NMIBLCA) and muscle-invasive BLCA (MIBLCA). The former is characterized by recurrence and progression while the latter is characterized by metastasis and unfavorable prognosis [2]. Despite the uplifting improvement in cancer therapy for the past two decades, including laparoscopic and robotic surgery, targeted therapy, and immune checkpoint inhibitor therapy, the 5-year survival rate of patients with MIBLCA remains unsatisfactory. Therefore, identifying novel biomarkers for predicting prognosis and response to therapeutic drug in BLCA is of considerable clinical meaning.

Peroxisome proliferator-activated receptors (PPARs) were the critical members of the steroid hormone receptor family. Meanwhile, PPARs were also a group of specific nuclear transcription factors activated by natural ligands (fatty acids and eicosanoids) and synthetic ligands (fibrates and thiazolidinediones) [3]. According to the various tissue distribution, metabolic patterns, and ligand specificity, PPARs were classified into three isotypes: PPAR*α*, PPAR*β*/*δ*, and PPAR*γ* [4]. PPAR*α* was mainly located in brown adipose tissue and liver and involved in eliminating cellular or circulating lipids [5, 6]. PPAR*γ* was primarily expressed in the adipose tissue and the immune system and correlated with adipose differentiation. PPAR*β*/*δ* was highest expressed in the gut, kidney, and heart and mainly implicated in lipid oxidation and cell proliferation. Accumulating studies have certified the crucial roles of PPARs in various biological processes including cell differentiation, apoptosis, inflammation, immune function, angiogenesis, metabolism, and carcinogenesis [3, 7–10]. Furthermore, several drugs that targeted PPARs have been applied in clinical trials [11]. However, the relationship between PPARs and outcomes of BLCA patients was still unknown.

In the present study, we investigated the expression and outcomes of PPARs through integrated bioinformatic approaches in BLCA. Subsequently, a PPAR-based signature was established for predicting the prognosis and drug sensitivity, and the predictive ability of this signature was validated in two external datasets. Furthermore, we also explored the correlation between the signature and clinical characteristics as well as tumor microenvironment. In addition, enrichment analysis was conducted to investigate the potential mechanisms of PPARs in BLCA. Finally, a nomogram was constructed to improve the clinical management of BLCA patients.

## 2. Materials and Methods

### 2.1. Data Acquisition

The RNA-sequencing data and clinical characteristic information of patients with BLCA were acquired from The Cancer Genome Atlas database (TCGA, https://gdc-portal.nci.nih.gov/). In addition, GSE13507 and GSE32894 were originated from the Gene Expression Omnibus database (GEO, https://www.ncbi.nlm.nih.gov/geo/) and served as the independent external validation datasets. 130 experimentally verified PPAR-targeted genes were obtained from the PPARgene database (http://www.ppargene.org/) [12].

### 2.2. Identification of Differentially Expressed PPAR Genes

The differential expression analysis was conducted with the edgeR package in R software (version R 3.6.1). Differentially expressed PPAR genes (DEPPARGs) were identified with the criterion of false discovery rate (FDR) < 0.05 and ∣log2 FC (fold change)  | >1 between BLCA and normal samples. Heatmap and volcano plot were used to display DEPPARGs. In addition, Gene Ontology (GO) analysis and Kyoto Encyclopedia of Genes and Genomes (KEGG) analysis were performed to investigate the potential functions of DEPPARGs by using the clusterProfiler packages in R software.

### 2.3. Identification of Candidate Small Molecule Drugs

To identify the potential small molecule drugs for treatments of patients with BLCA, the Connectivity Map database (CMAP, https://portals.broadinstitute.org/cmap/) was performed to select the candidate drugs. The enrichment score was used to evaluate the effect of a drug, and the negative score indicated that a drug might have antitumor activity.

### 2.4. Establishment and Validation of the PPAR-Related Prognostic Signature

To identify the DEPPARGs associated with overall survival (OS), univariate Cox regression analysis was performed to explore the relationship between DEPPARGs and prognosis of BLCA patients in the TCGA dataset. All the DEPPARGs with *P* value < 0.05 were identified as candidate genes for subsequent analyses. Then, the least absolute shrinkage and selection operator (LASSO) analysis was used to shun the overfitting and identify optimal prognostic DEPPARGs. Finally, multivariate Cox regression analysis was conducted to establish an optimized risk score (PPARscore). The PPARscore of patients with BLCA was calculated by the following formula: PPARscore = ∑_*i*_^*n*^X_*i*_ × Y_i_ (where *Y* represented the mRNA expression of gene and *X* represented the coefficient of the relevant gene from the multivariate Cox analysis). Patients were classified into high- and low-risk groups based on the median risk score. Survival analyses were conducted to evaluate the difference of prognosis among different groups by R packages (survival and survminer) in R. In addition, a receiver operating characteristic (ROC) curve was performed to test the predictive performance of the signature by using the survivalROC package. Moreover, principal component analysis (PCA) and *t*-distributed stochastic neighbor embedding (*t*-SNE) were executed to investigate the distribution characteristics of patients among two groups. In addition, the predictive power of our constructed signature was verified in the two external validation datasets (GSE13507 and GSE32894) by using the same approach.

### 2.5. Gene Set Enrichment Analysis and Immune Infiltration Analyses

Gene set enrichment analysis (GSEA) was used to explore the underlying biological mechanisms of the PPAR-based signature with the criterion of *P* value < 0.05 and FDR < 0.25. Given the importance of the tumor immune microenvironment, the ESTIMATE algorithm was conducted to evaluate the stromal score, ESTIMATE score, and immune score among two groups. In addition, the CIBERSORT algorithm was performed to explore the immune cell infiltration levels of 22 distinct leukocyte subsets among different groups. Furthermore, we also investigated the correlation between PPARscore and key immune checkpoints (PD-1, PD-L1, CTLA4, LAG3, HAVCR2, and TIGIT). *P* values < 0.05 were considered as statistical criteria.

### 2.6. Chemotherapy Sensitivity Prediction

To assess the difference of chemotherapy sensitivity between different groups, we used the GDSC database to estimate the half-maximal inhibitory concentration (IC50) of chemotherapy drugs for predicting the sensitivity of chemotherapy drugs by using the package (pRRophetic). *P* values < 0.05 were considered statistically significant.

### 2.7. Construction of a Nomogram

Univariate and multivariate cox regression analyses were conducted to investigate whether the PPAR-based signature was an independent prognosis factor in patients with BLCA. Furthermore, we also categorized patients into various subgroups stratified by clinical features, and the Kaplan-Meier curves were conducted in each subgroup to further test the predictive power of the PPAR-based signature in predicting prognosis. Based on the results of multivariate analysis, a nomogram consisting of risk score and several clinical features for predicting the over survival of 3 and 5 years was established. The calibration curve was used to evaluate the accuracy of survival prediction in the nomogram.

## 3. Results

### 3.1. Identification of Differentially Expressed Genes

**Figure 1:**
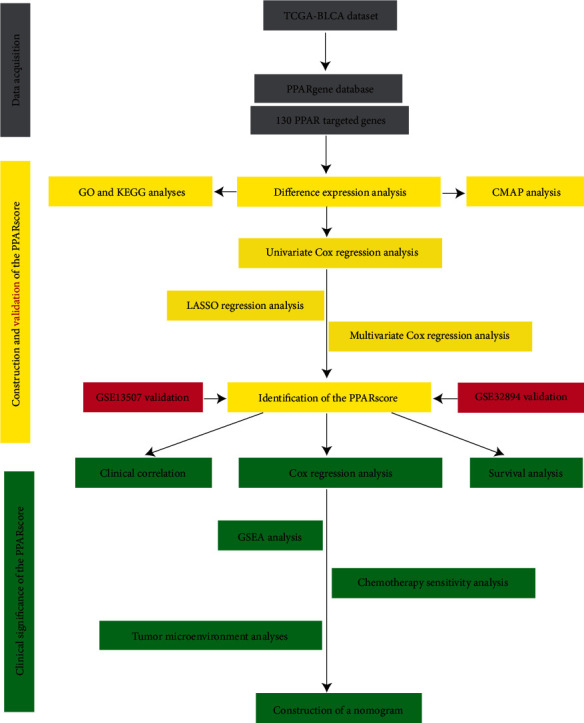
The flow chart of the PPAR signature in predicting survival of BLCA.

**Figure 2:**
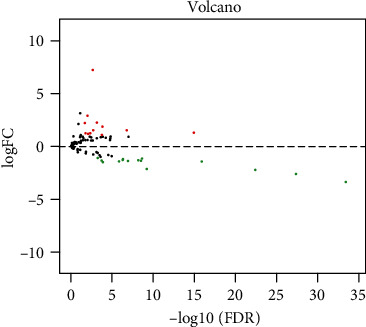
Volcano map showed the differentially expressed PPAR-targeted genes (DEPPARGs). The red plots represented the upregulated genes, the blue plots represented the underregulated genes, and the black plots represented no statistically differentially expressed genes with the criterion of FDR < 0.05 and ∣log2 FC | >1. FDR: false discovery rate; FC: fold change.

Figure 1 displays the procedure of our study. The mRNA expression profile of 130 PPAR target genes between BLCA samples (*n* = 414) and normal bladder samples (*n* = 19) was obtained from the TCGA dataset. A total of 27 DEPPARGs were identified with the threshold of FDR < 0.05 and ∣log2 FC | >1, including 15 downregulated and 12 upregulated genes (Figure 2). The volcano map was used to exhibit the expression profile of DEPPARGs. To further expound the potential mechanisms of DEPPARGs, GO and KEGG analyses were performed with the 27 DEPPARGs. GO analysis indicated that DEPPARGs were mainly implicated in the regulation of lipid metabolic process, triglyceride metabolic process, acylglycerol metabolic process, neutral lipid metabolic process, lipid localization, and reactive oxygen species metabolic process (Supplementary Figure 1(a)). The result of KEGG analysis revealed that DEPPARGs were mainly involved in the PPAR signaling pathway, cholesterol metabolism, ovarian steroidogenesis, platinum drug resistance, and microRNAs in cancer (Supplementary Figure 1(b)), which suggested that DEPPARGs might function as the crucial role in the tumorigenesis, progression, and drug resistance of BLCA.

### 3.2. Small Molecular Drugs

To further enhance the therapeutic efficacy of BLCA, the CMAP database was performed to identify candidate drugs based on the DEPPARGs. The eleven small molecular drugs with anticancer activity were identified (Table 1). These drugs (vorinostat, cinchonine, helveticoside, lanatoside C, tiapride, idoxuridine, niclosamide, ampicillin, epitiostanol, pyrimethamine, and cephaeline) might alleviate the progression of BLCA and serve as novel potential targeted drugs for BLCA treatment.

### 3.3. Construction of a Prognostic PPAR Signature

**Figure 3:**
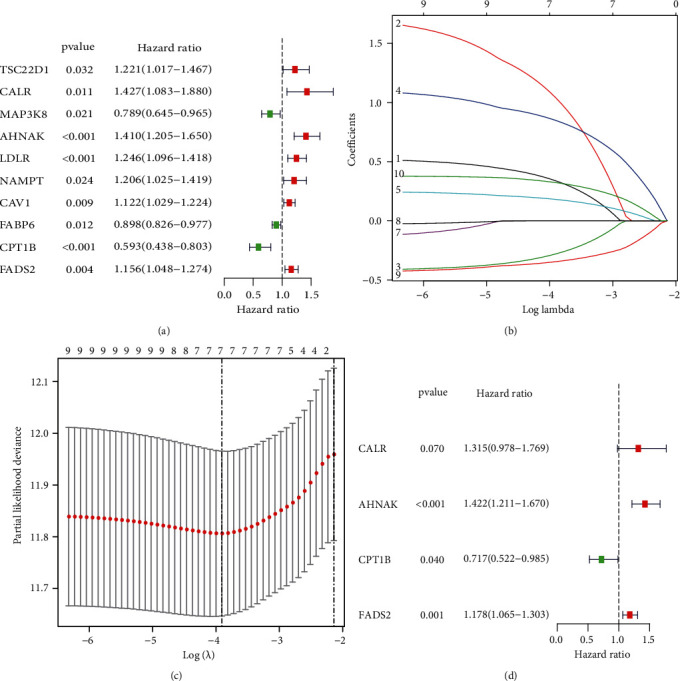
Identification of DEPPARGs correlated with prognosis in the TCGA dataset. (a) Identification of the prognostic DEPPARGs by univariate Cox regression analysis; (b) the coefficient profile of 9 prognostic genes by LASSO regression analysis; (c) tenfold cross-validation for tuning parameter selection in the LASSO analysis; (d) identification of 4 prognostic DEPPARGs by multivariate Cox regression analysis.

**Figure 4:**
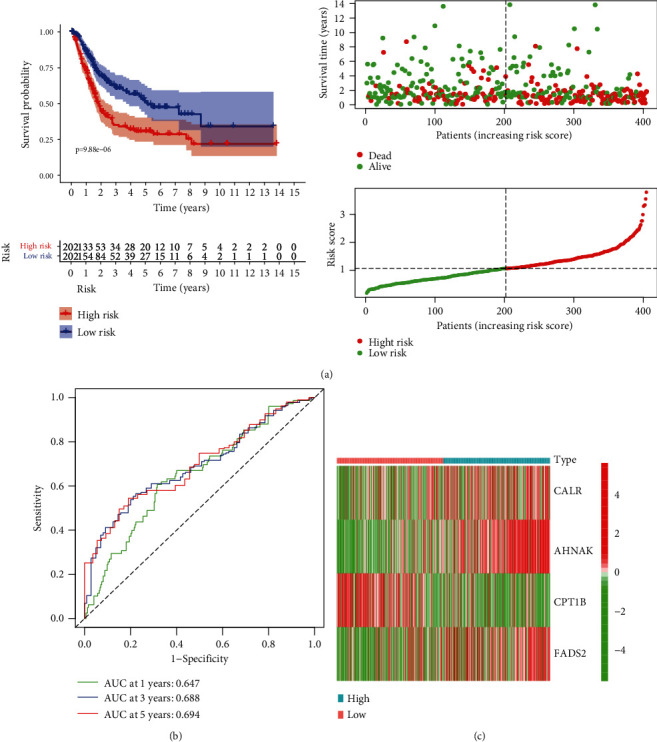
Construction of the PPAR signature correlated with prognosis in the TCGA dataset. (a) Kaplan-Meier curve showed that low-risk patients had better survival compared with the high-risk patients. The distribution of overall survival between the high-risk group and the low-risk group. The distribution of PPARscore between the high-risk group and the low-risk group; (b) time-independent receiver operating characteristic (ROC) analysis for evaluating the predictive performance of PPARscore; (c) heatmap showed the expression patterns of 4 genes between the high- and low-risk groups.

Based on the 27 DEPPARGs, Cox and LASSO regression analyses were implemented to identify DEPPARGs correlated with OS in the TCGA dataset. First, ten DEPPARGs exhibited fairly correlation with the outcomes of patients with BLCA via univariate Cox regression analysis (Figure 3(a)). Then, to guarantee the reliability of ten prognostic genes, LASSO regression analysis was performed to further screen DEPPARGs without the overfitting (Figures 3(b) and 3(c)). Finally, based on the result of multivariate Cox regression analysis, four DEPPARGs, including CALR, FADS2, CPT1B, and AHNAK, were identified and applied to establish a prognostic signature (Figure 3(d)). We developed a four gene-based PPARscore as follows: PPARscore = (0.2739 × CALR expression) + (0.352 × AHNAK expression) + (−0.3324 × CPT1B expression) + (0.164 × FADS2 expression). Patients were then categorized into high- and low-risk groups in accordance with the median PPARscore. PCA and *t*-SNE analyses also displayed the various dimensions between the high-risk group and the low-risk group (Supplementary Figures 2(a) and 2(b)). The prognosis of patients in the low-risk group was significantly superior to those in the high-risk group (*P* < 0.05) (Figure 4(a)). Time-dependent ROC analysis suggested that the AUC values for 1-, 3-, and 5-year survival of PPARscore in the TCGA dataset were 0.647, 0.688, and 0.694, respectively (Figure 4(b)). These results indicated that the PPAR signature might have a certain applicability in predicting the outcomes of patients with BLCA. Additionally, the heatmap of the expression profiles of four genes showed that CALR, AHNAK, and FADS2 were highly expressed in the high-risk group, while CPT1B was elevated in the low-risk group (Figure 4(c)).

### 3.4. Validation of the PPAR Signature

**Figure 5:**
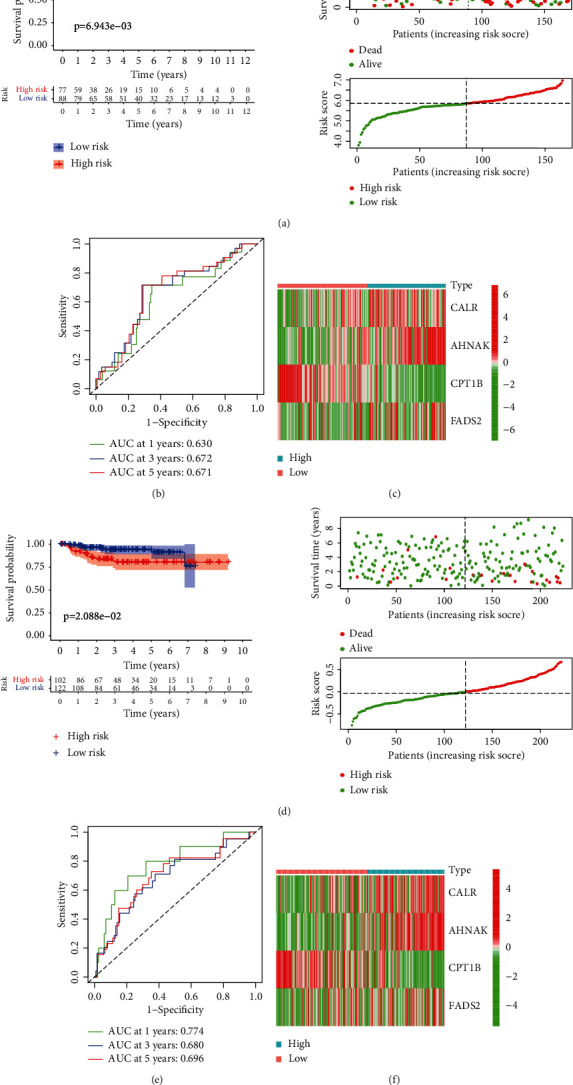
Validation of the PPAR signature in the GEO dataset. (a–e) Kaplan-Meier curve showed that low-risk patients had better survival compared with the high-risk patients. The distribution of overall survival between the high-risk group and the low-risk group. The distribution of PPARscore between the high-risk group and the low-risk group ((a) GSE13507; (e) GSE32894); (b–f) time-independent receiver operating characteristic (ROC) analysis for evaluating the predictive performance of PPARscore ((b) GSE13507; (f) GSE32894); (c–g) heatmap showed the expression patterns of 4 genes between the high- and low-risk groups ((c) GSE13507; (g) GSE32894). The blue box represented the GSE13507 dataset; the red box represented the GSE32894 dataset.

In GSE13507, survival time and status from 165 patients with BLCA were applied to validate our constructed signature. The PPARscore of each patient was generated with the same approach as before, and patients were classified into high- and low-risk groups in accordance with the median PPARscore. PCA and *t*-SNE analyses displayed the diverse dimensions between the high-risk group and the low-risk group (Supplementary Figures 2(c) and 2(d)). *K*-*M* curve analysis indicated that the prognosis of patients in the low-risk group was significantly superior to those in the high-risk group (*P* < 0.05) (Figure 5(a)). Time-dependent ROC analysis suggested that the AUC values for 1-, 3-, and 5-year survival of PPARscore in the TCGA dataset were 0.630, 0.672, and 0.671, respectively (Figure 5(b)). Similarly, in GSE32894, 224 patients containing survival time and status were served as another external validation dataset. The results of GSE32894 were also consistent with the previous results (Figures 5(e) and 5(f)). Taken together, all these results revealed that the PPAR signature might serve as a potential biomarker for predicting the outcomes of BLCA patients.

### 3.5. GSEA

To further illustrate the molecular mechanisms of PPAR signature, GSEA analysis was conducted. The results of GSEA analysis indicated that focal adhesion, pathways in cancer, GAP junction, chemokine signaling pathway, WNT signaling pathway, TGF-*β* signaling pathway, steroid biosynthesis, bladder cancer, MAPK signaling pathway, and calcium signaling pathway were mainly enriched in the high-risk group, suggesting that patients of high-risk groups were notably related to cancer-related signaling pathway, while oxidative phosphorylation and cardiac muscle contraction were highly enriched in the low-risk group (Supplementary Figure 3).

### 3.6. Immune Landscape of the PPAR Signature

**Figure 6:**
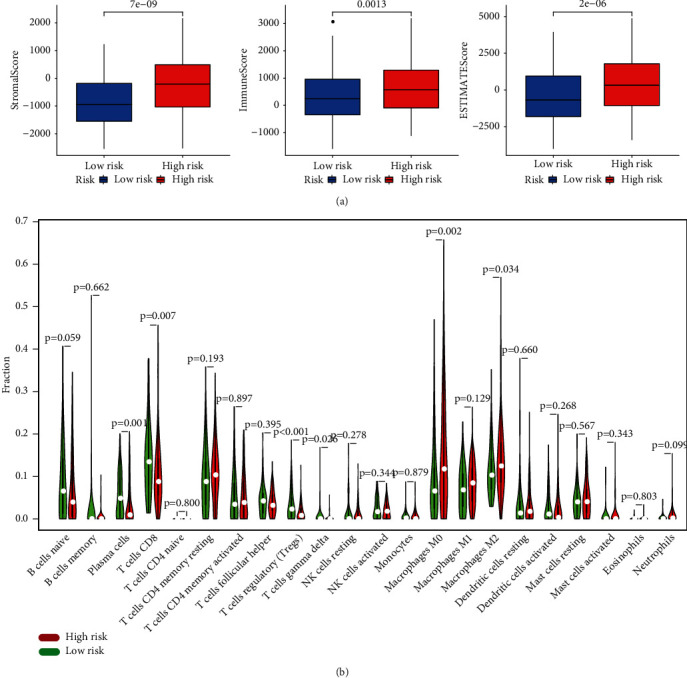
The relationship between PPARscore and tumor microenvironment. (a) Boxplot showed the relative expressions of ESTIMATE score, immune score, and stromal score between high- and low-risk groups by the ESTIMATE algorithm; (b) violin plot compared the expressions of 22 immune cells infiltrating between the high- and low-risk groups by the CIBERSORT algorithm. The red signified the high-risk groups, and the blue signified the low-risk groups.

To explore whether PPAR signature could illustrate the characteristic of tumor immune microenvironment, ESTIMATE and CIBERSORT algorithms associated with immune cell infiltration were conducted. The results of ESTIMATE suggested that patients with high PPARscore displayed a higher immune score, stromal score, and ESTIMATE score than patients with low PPARscore (Figure 6(a)), which indicated that PPARscore might be correlated with the tumor microenvironment. Furthermore, the results of CIBERSORT revealed that the proportions of CD8+ T cell, Tregs, plasma cell, and T cells gamma delta were obviously higher in patients with low PPARscore, while the proportions of M2 macrophages and M0 macrophages were remarkably higher in patients with high PPARscore (Figure 6(b) and Supplementary Figure 4). In addition, we also compared the expression of key immune checkpoints between two groups and found that immune checkpoints (PD-L1, LAG3, TIGIT, and HAVCR2) were upregulated in the high-risk group, while the expressions of CTLA4 and PD-1 were no different among two groups (Supplementary Figure 5). All these results uncovered that the PPAR signature might be implicated in the tumorigenesis and progression of BLCA via regulating the infiltrating distribution of immune cells.

### 3.7. Chemotherapeutic Response Analysis

**Figure 7:**
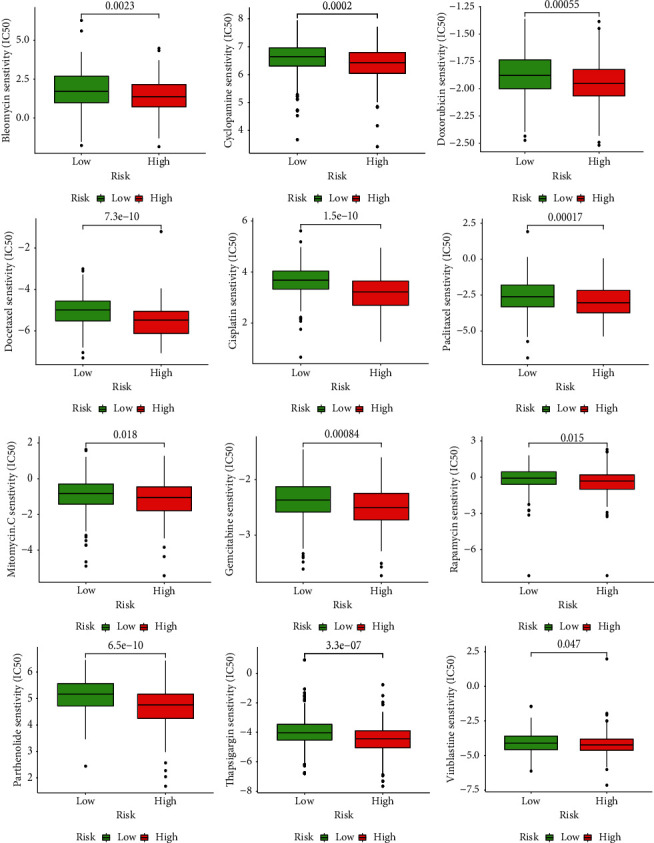
Comparison of the estimated IC50 values of Bleomycin, Mitomycin C, Gemcitabine, Cyclopamine, Docetaxel, Cisplatin, Paclitaxel, Rapamycin, Parthenolide, Thapsignargin, Vinblastine, and Doxorubicin in the high- and low-risk BLCA samples by using the GDSC database.

To improve the therapeutic effect of BLCA patients, we further investigated whether our PPAR signature could predict the sensitivity to several common chemotherapy drugs between two groups. The results of GDSC database analysis suggested that IC50 values of chemotherapy drugs including Bleomycin, Mitomycin C, Gemcitabine, Cyclopamine, Docetaxel, Cisplatin, Thapsignargin, Paclitaxel, Rapamycin, Parthenolide, Vinblastine, and Doxorubicin were elevated in patients with low PPARscore compared to those with high PPARscore, which indicated that patients with high PPARscore were much more sensitive to these chemotherapy drugs (Figure 7).

### 3.8. Relationship between PPAR Signature and Clinical Characteristics

**Figure 8:**
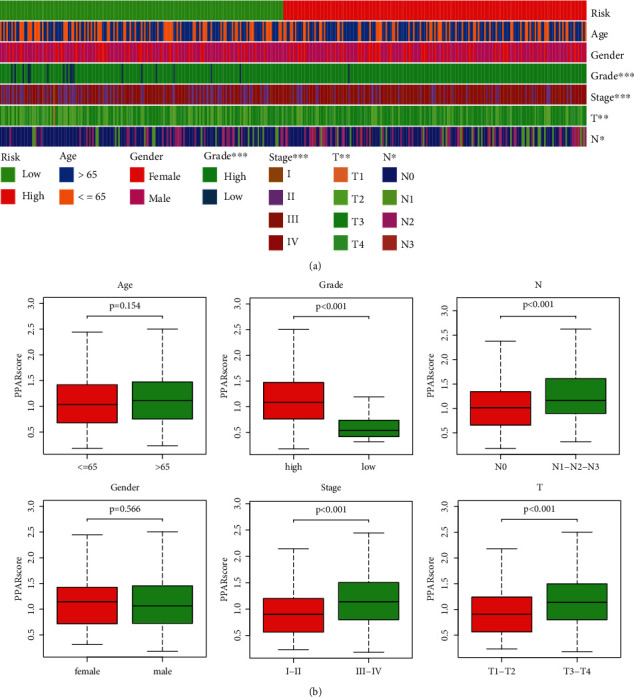
The correlation between the risk scores and clinicopathological factors. (a) Heatmap showed the relative expression of the risk score in BLCA patients at various clinical features, including age, gender, tumor grade, TNM stage, T stage, and N stage. ^∗∗∗^*P* < 0.001<^∗∗^*P* < 0.01<^∗^*P* < 0.05; (b) boxplot showed the relative expression of the risk score in BLCA patients at subgroups stratified by age, gender, tumor grade, TNM stage, T stage, and N stage.

To improve the clinical management of BLCA patients, we also explored the correlation between PPARscore and clinical characteristics in the TCGA dataset. Heatmap displayed the distributions of clinical characteristics including tumor grade, tumor stage, gender, age, N stage, and T stage between two groups, and obvious differences were observed in tumor grade, tumor stage, T stage, and N stage (Figure 8(a)). In addition, the boxplot exhibited the significant correlation between PPARscore with the poorer clinical characteristics (T3-T4 stage, N1-N2-N3 stage, stage III–IV, and grade high) (Figure 8(b)). These results suggested that PPARscore might be related to the progression of BLCA.

### 3.9. Construction of a Nomogram

**Figure 9:**
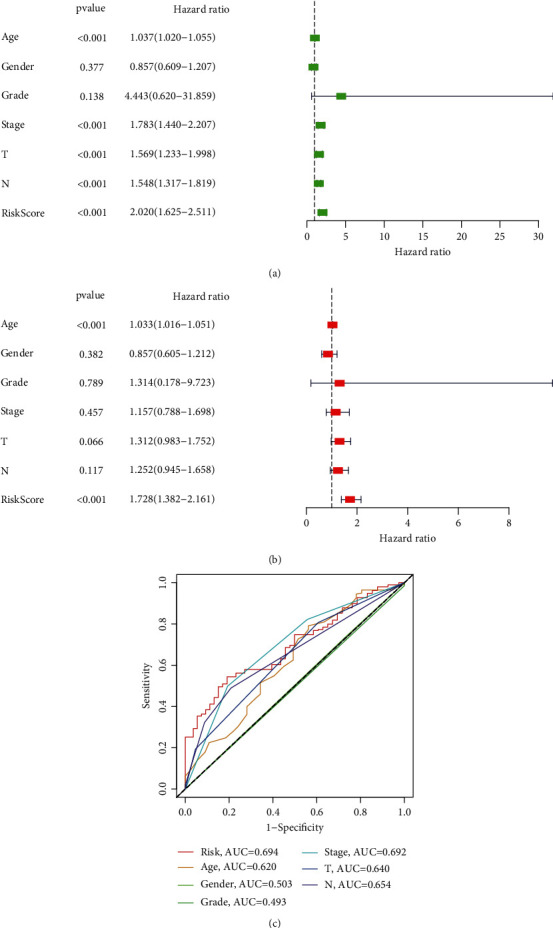
The PPAR signature was an independent prognostic factor for BLCA in the TCGA set. (a) The univariate Cox analysis for evaluating the independent prognostic value of PPAR signature; (b) multivariate Cox analysis for evaluating the independent prognostic value of PPAR signature; (c) ROC curve analyses of the clinical characteristics and risk score.

**Figure 10:**
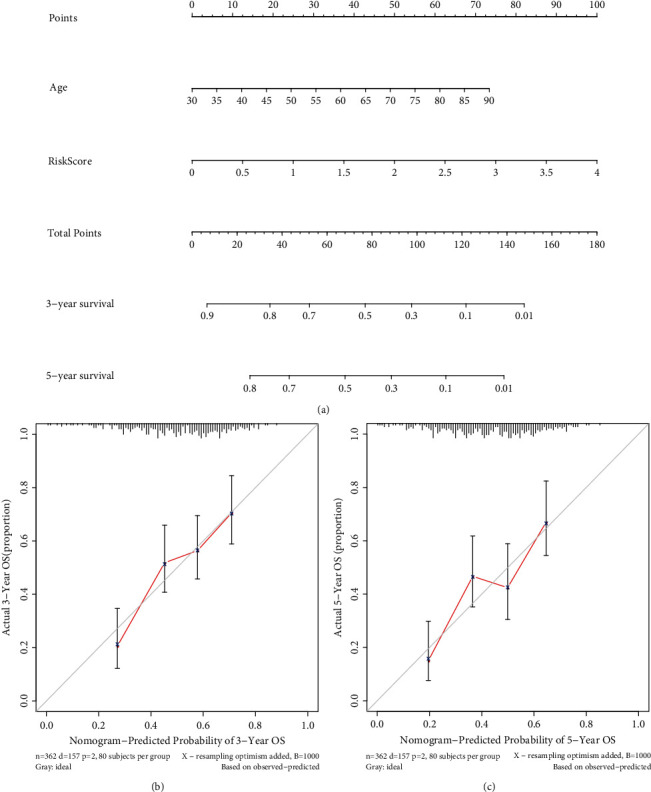
Establishment of the nomogram in the TCGA dataset. (a) Nomogram based on the PPARscore and age; (b) the 3-year calibration plot for the nomogram; (c) the 5-year calibration plot for the nomogram.

Univariable and multivariable Cox analyses were applied to further explore whether PPARscore could be an independent prognostic indicator for BLCA patients. The result of univariable Cox analysis indicated that PPARscore, tumor stage, tumor grade, T stage, and N stage were obviously correlated with outcomes of BLCA patients (Figure 9(a)). The result of multivariable Cox analysis showed that PPARscore and age were still associated with outcomes of BLCA patients, which suggested that the PPARscore model could be an independent prognostic factor of BLCA patients (Figure 9(b)). Furthermore, multiparameter ROC curve analyses showed that the AUC value of PPARscore was 0.694 (Figure 9(c)), which suggested that PPARscore was superior to traditional clinical prognostic indicators in predicting outcomes. In addition, the results of subgroup analyses stratified by various clinical characteristics indicated an obviously shorter survival probability in patients with high PPARscore among various clinical characteristics except T1-T2 stage, and stage I-II subgroups (Supplementary Figure 6). Based on the result of multivariable Cox analysis, PPARscore and age were incorporated to construct a nomogram to preferably predict the survival ability of 3 and 5 years (Figure 10(a)). The calibration curves suggested that the nomogram exhibited the well performance in forecasting the prognosis (Figures 10(b) and 10(c)).

## 4. Discussion

In the current study, the expression pattern of PPAR-targeted genes could predict the outcomes of in BLCA, and four genes were applied to further construct and validate a prognostic PPARscore. Furthermore, PPARscore was also available in predicting sensitivity to chemotherapy drugs. In addition, PPARscore was also correlated with adverse clinical features and immune cells. In GSEA analysis, PPARscore was observed to be implicated in various signaling pathways correlated with tumorigenesis.

Four genes were incorporated in our signature (CPT1B, CALR, FADS2, and AHNAK). Carnitine palmitoyltransferase 1B (CPT1B), a crucial enzyme of long-chain fatty acid *β*-oxidation and also a member of the PPAR pathway, has been found to be underexpressed in high-grade BLCA. In addition, the overexpression of CPT1B could inhibit the proliferation and metastasis of BLCA cells by accelerating fatty acid metabolism and reducing epithelial-mesenchymal transition (EMT) [13]. Calreticulin (CALR), a crucial member of endoplasmic reticulum (ER) chaperones, was positively related to superior prognosis owing to the activation of anticancer immune in various cancers [14–17]. CALR overexpression was obviously related to advanced grade and poor prognosis in BLCA [18, 19]. Fatty acid desaturase 2 (FADS2), a key enzyme of polyunsaturated fatty acid (PUFA) metabolism, was involved in multiple diseases including cancer. FADS2 was adversely related to prognosis in BLCA by bioinformatic analysis [20]. In addition, Jiang et al. also reported that FADS2 might serve as a ferroptosis suppressor [21]. The aberrant expression of AHNAK has been reported in various cancer [22–26]. For example, AHNAK overexpression inhibited the TNBC cell proliferation and lung metastasis by partly regulating the Wnt/*β*-catenin signaling pathway.

Subsequently, we performed GSEA analysis to further disclose the mechanism of PPAR signature in BLCA. The results confirmed that PPAR signature was involved in cancer-related pathways including focal adhesion, pathways in cancer, WNT signaling pathway, TGF-*β* signaling pathway, bladder cancer, and MAPK signaling pathway. Therefore, PPAR signature could serve as a predictor for BLCA prognosis and might play a critical role in BLCA biology.

Numerous evidences have proven the crucial influence of tumor microenvironment upon the progression, prognosis, and therapy in BLCA. The higher infiltration level of CD8+ T cells was positively correlated with better prognosis [27–29]. Increasing evidence suggests that M2 macrophages could accelerate the malignant progression and distant metastasis [30, 31]. Furthermore, M2 macrophages were also correlated with the immunosuppressive microenvironment and unfavorable prognosis [32, 33]. T cells gamma delta, also known as *γδ* T cells, were characterized with the antigen specificity and NK-like cytotoxicity. *γδ* T cells can recognize and present tumor antigen in a major histocompatibility complex- (MHC-) independent manner, and activated *γδ* T cells could enhance the antitumor activity of adaptive immune cells [34, 35]. In addition, *γδ* T cells also have been reported to be related to favorable prognosis [36]. Nevertheless, some studies have reported that *γδ* T cells facilitated tumor progression by promoting angiogenesis, recruiting inhibitory cells, and enhancing the apoptosis of antitumor immune cells [37–39]. *γδ* T cells played an important role in antitumor activity of intravesical bacillus Calmette-Guérin (BCG) against BLCA [40, 41]. *γδ* T cells also can heighten the carboplatin-induced cytotoxicity to BLCA [42]. Our results also suggested that PPAR signature displayed the strong correlation with tumor microenvironment as well as immune cell infiltration. Furthermore, the results showed that CD8+ T cell, Tregs, M0 and M2 macrophages, plasma cell, and T cells gamma delta were significantly distinct between high- and low-risk groups. Patients with high PPARscore had more proportions of M0 and M2 macrophages while patients with low PPARscore had more proportions of CD8+ T cell, plasma cell, and T cells gamma delta. In addition, immune checkpoints including PD-L1, HAVCR2, TIGIT, and LAG3 in the high-risk group were also higher than those in the low-risk group, which indicated that patients in the high-risk group might belong to the “hot” tumor that was tended to benefit from immune checkpoint inhibitor therapy [43]. Furthermore, based on the results of the GDSC database, patients in the high-risk group also might benefit from chemotherapy drugs including Bleomycin, Mitomycin C, Gemcitabine, Cyclopamine, Docetaxel, Cisplatin, Paclitaxel, Rapamycin, Parthenolide, Vinblastine, and Doxorubicin.

We found that eleven small molecule drugs, such as vorinostat, cinchonine, helveticoside, lanatoside C, tiapride, idoxuridine, niclosamide, ampicillin, epitiostanol, pyrimethamine, and cephaeline, could improve the therapeutic effect of BLCA patients. Pyrimethamine, an antimalarial drug, has been observed to inhibit the proliferation and induce the apoptosis in various cancers [44–46].

Of course, there were also several disadvantages in our study. On one hand, the prognostic PPAR signature was constructed and validated only by public database and retrospective research and required to be verified through a prospective trial. On the other hand, the molecular mechanisms of PPAR signature in BLCA should be further validated by in vivo or in vitro experiments.

## 5. Conclusion

We comprehensively explored the clinical significance of PPAR-targeted genes and constructed and validated a novel PPAR signature, which showed the excellent performance in predicting prognosis and chemotherapy sensitivity of BLCA patients. Furthermore, we also investigated the correlation between PPAR signature and tumor microenvironment. Finally, several small molecule drugs were identified for the treatments of BLCA patients. All these results uncovered the crucial role of PPAR in BLCA progression and provided novel directions for BLCA therapeutic intervention.

## Figures and Tables

**Table 1:** The 11 small-molecule drugs of CMP database analysis.

cmap name	Mean	*n*	Enrichment	*P* value	Percent nonnull
Vorinostat	-0.766	2	-0.995	0.00008	100
Cinchonine	-0.697	2	-0.983	0.00058	100
Helveticoside	-0.83	2	-0.982	0.0007	100
Lanatoside C	-0.818	2	-0.981	0.00082	100
Idoxuridine	-0.64	2	-0.963	0.00304	100
Niclosamide	-0.621	2	-0.959	0.00368	100
Ampicillin	-0.607	2	-0.958	0.0038	100
Epitiostanol	-0.595	2	-0.952	0.00497	100
Tiapride	-0.652	2	-0.972	0.00159	100
Pyrimethamine	-0.57	2	-0.935	0.00891	100
Cephaeline	-0.521	2	-0.879	0.00347	100

## Data Availability

The RNA-sequencing data and clinical characteristic information of patients with BLCA were acquired from the TCGA database (https://gdc-portal.nci.nih.gov/). In addition, validation datasets (GSE13507 and GSE32894) were originated from the GEO database (https://www.ncbi.nlm.nih.gov/geo/). PPAR-targeted genes were obtained from the PPARgene database (http://www.ppargene.org/).

## Supplementary Materials

Supplementary 1 Supplementary Figure 1: functional enrichment analyses of differentially expressed PPAR-targeted genes (DEPPARGs). (A) GO analysis; (B) KEGG pathway analysis. GO: Gene Ontology; KEGG: Kyoto Encyclopedia of Genes and Genomes.

Supplementary 2 Supplementary Figure 2: PCA and *t*-SNE analyses of BLCA patients between the high- and low-risk groups. (A) PCA analysis in the TCGA dataset; (B) *t*-SNE analysis in the TCGA dataset; (C) PCA analysis in the GSE13507 dataset; (D) *t*-SNE analysis in the GSE13507 dataset; (E) PCA analysis in the GSE32894 dataset; (F) *t*-SNE analysis in the GSE32894 dataset. PCA: principal component analysis; *t*-SNE: *t*-distributed stochastic neighbor embedding.

Supplementary 3 Supplementary Figure 3: the potential pathways by gene set enrichment analysis (GSEA) between the high- and low-risk groups.

Supplementary 4 Supplementary Figure 4: the correlation between the risk score and immune cell infiltration, including M0 macrophage, M2 macrophage, CD8+ T cells, Tregs, gamma delta T cells, and plasma cells.

Supplementary 5 Supplementary Figure 5: the expression of key immune checkpoint genes between high- and low-risk groups.

Supplementary 6 Supplementary Figure 6: Kaplan-Meier curve analyses showed the overall survival stratified by age, gender, TNM stage, grade, N stage, and T stage between the high-risk and low-risk groups.
