# Supplementary figures and images for: Identification of a Novel PPAR Signature for Predicting Prognosis, Immune Microenvironment, and Chemotherapy Response in Bladder Cancer
Source: PPAR Res. 2021 Dec 30;2021:7056506. doi: 10.1155/2021/7056506 (PMC8749226; doi:10.1155/2021/7056506)

**A**

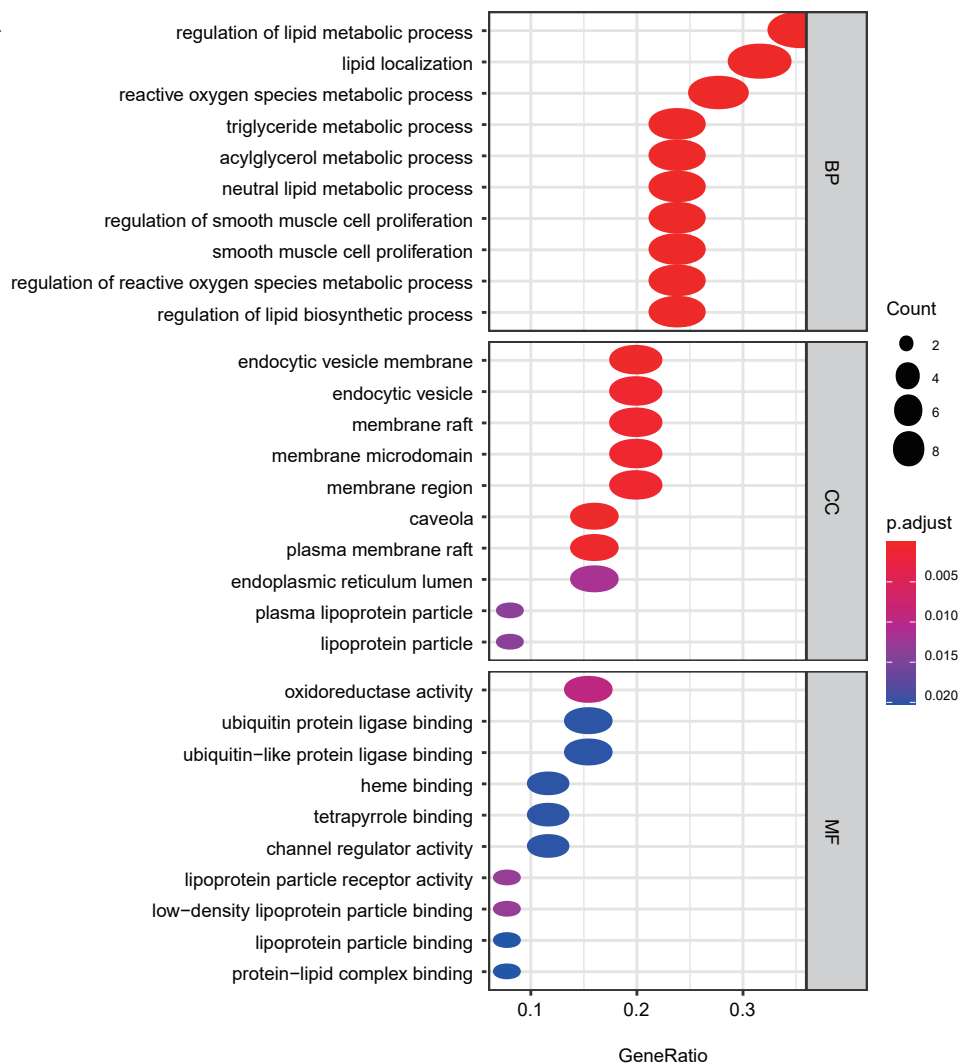

**B**

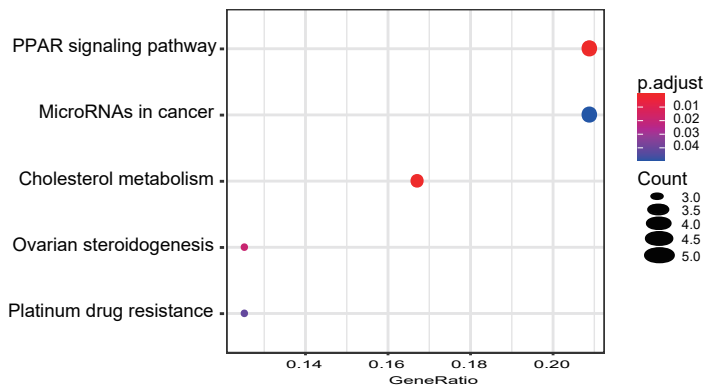

Supplement: Supplementary 1 — Supplementary Figure 1: functional enrichment analyses of differentially expressed PPAR-targeted genes (DEPPARGs). (A) GO analysis; (B) KEGG pathway analysis. GO: Gene Ontology; KEGG: Kyoto Encyclopedia of Genes and Genomes. [file 7056506.f1.pdf]

**A**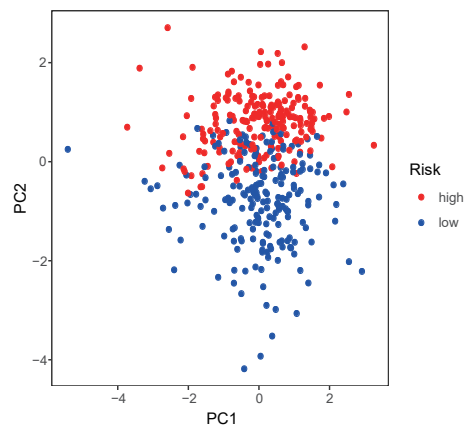**B**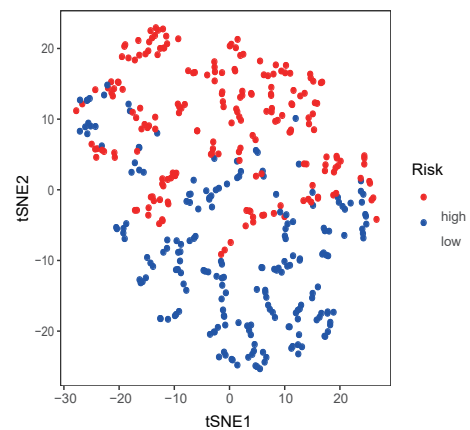**C**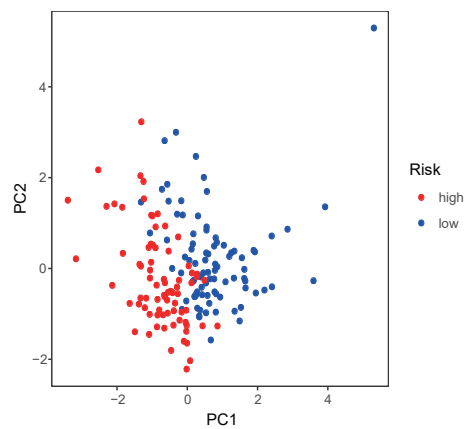**D**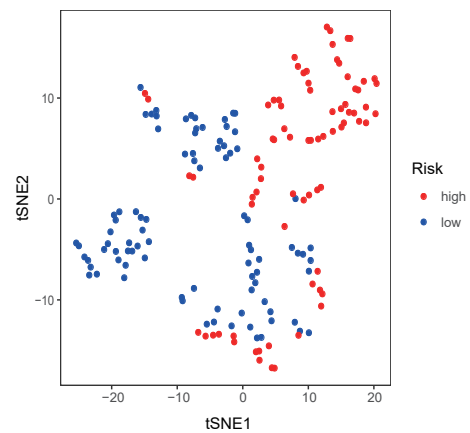**E**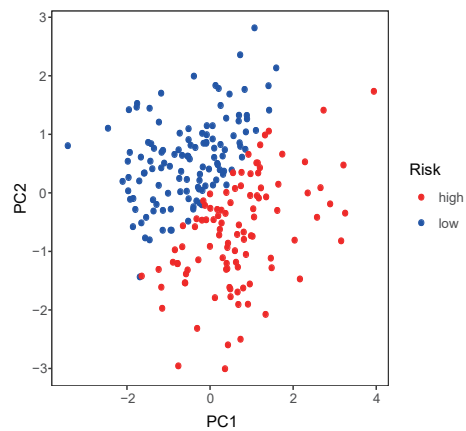**F**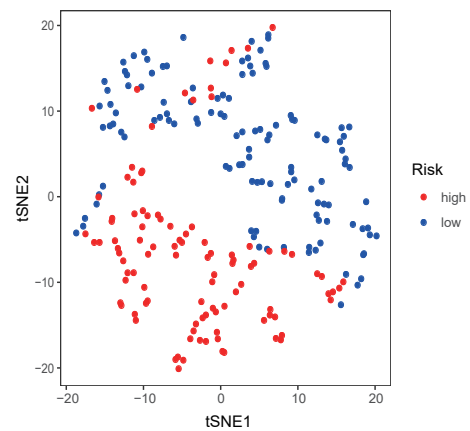

Supplement: Supplementary 2 — Supplementary Figure 2: PCA and t-SNE analyses of BLCA patients between the high- and low-risk groups. (A) PCA analysis in the TCGA dataset; (B) t-SNE analysis in the TCGA dataset; (C) PCA analysis in the GSE13507 dataset; (D) t-SNE analysis in the GSE13507 dataset; (E) PCA analysis in the GSE32894 dataset; (F) t-SNE analysis in the GSE32894 dataset. PCA: principal component analysis; t-SNE: t-distributed stochastic neighbor embedding. [file 7056506.f2.pdf]

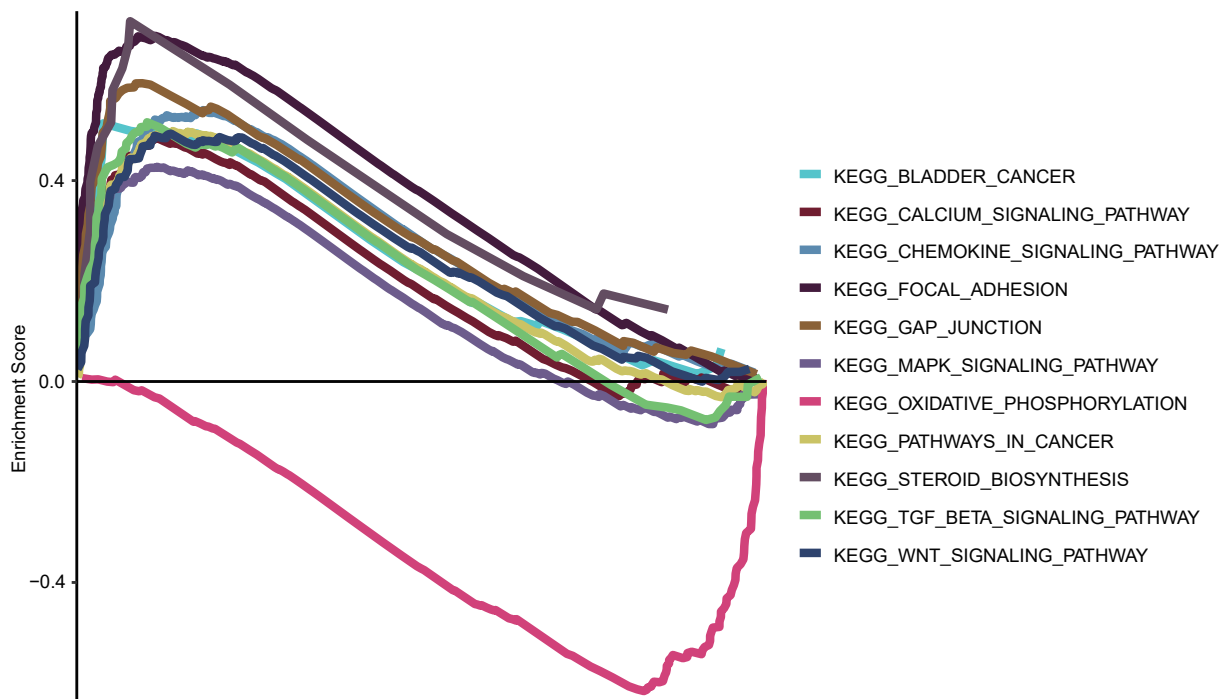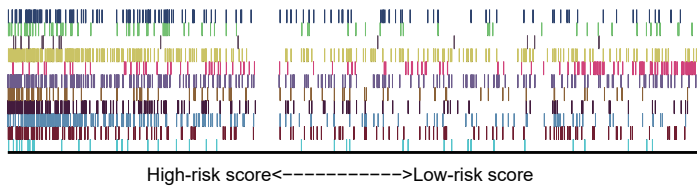

Supplement: Supplementary 3 — Supplementary Figure 3: the potential pathways by gene set enrichment analysis (GSEA) between the high- and low-risk groups. [file 7056506.f3.pdf]

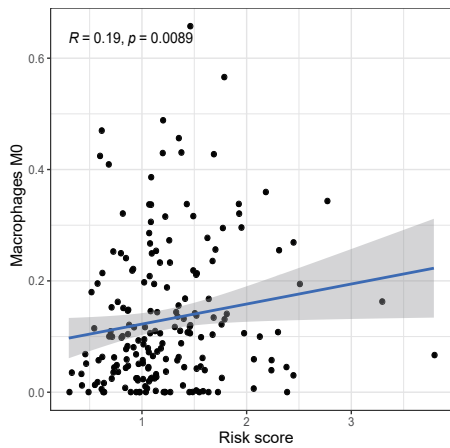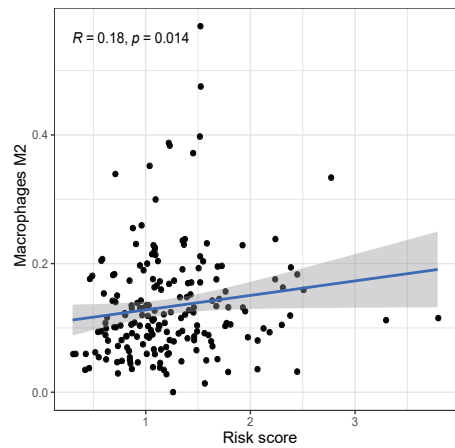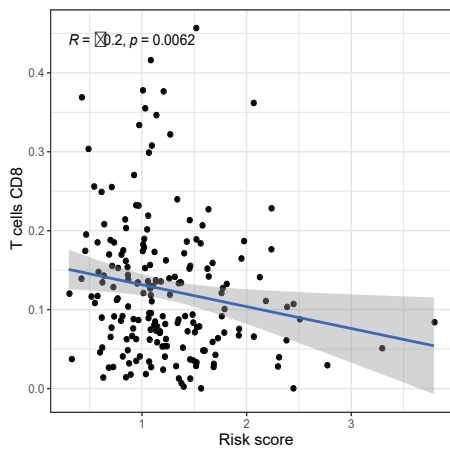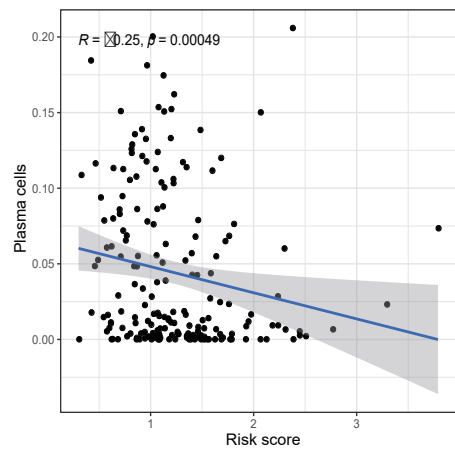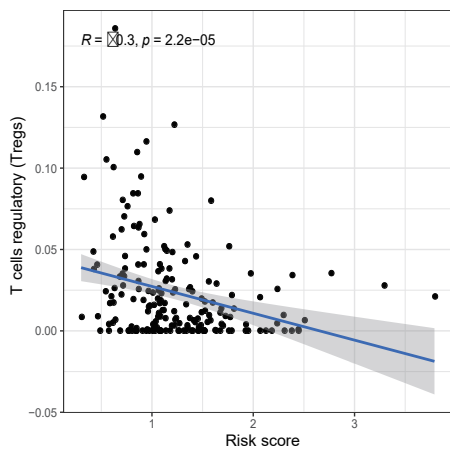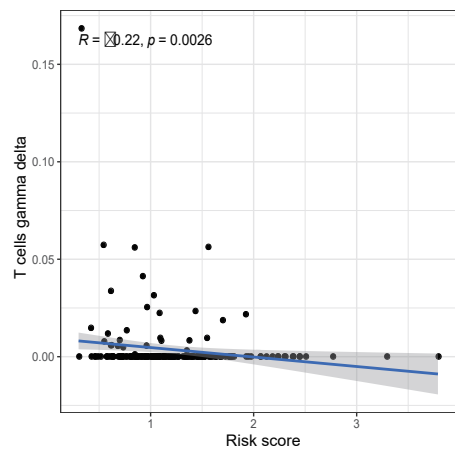

Supplement: Supplementary 4 — Supplementary Figure 4: the correlation between the risk score and immune cell infiltration, including M0 macrophage, M2 macrophage, CD8+ T cells, Tregs, gamma delta T cells, and plasma cells. [file 7056506.f4.pdf]

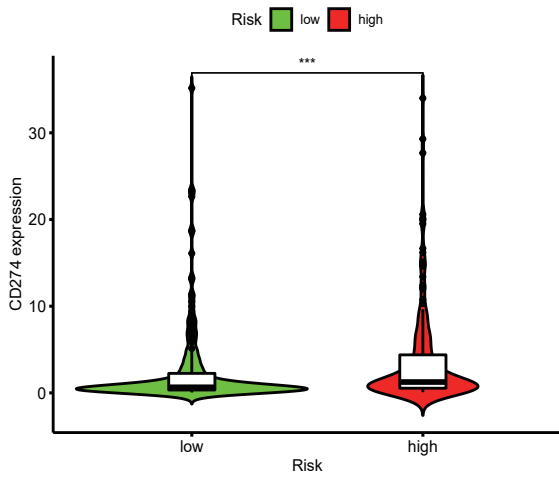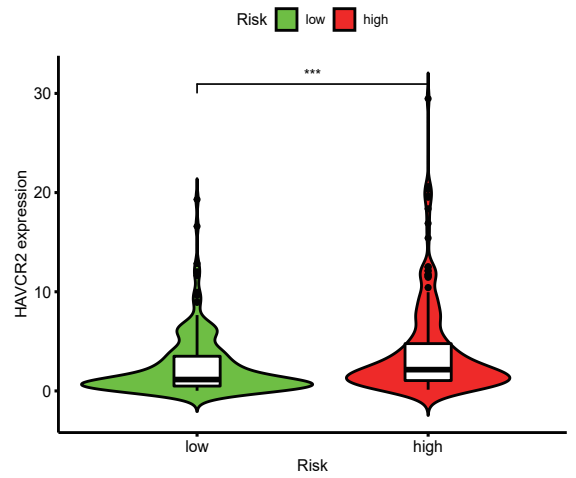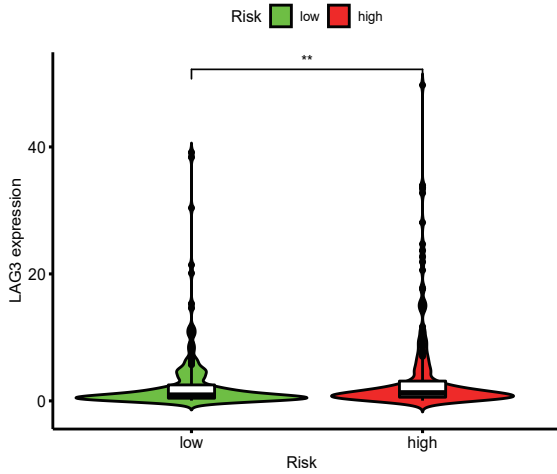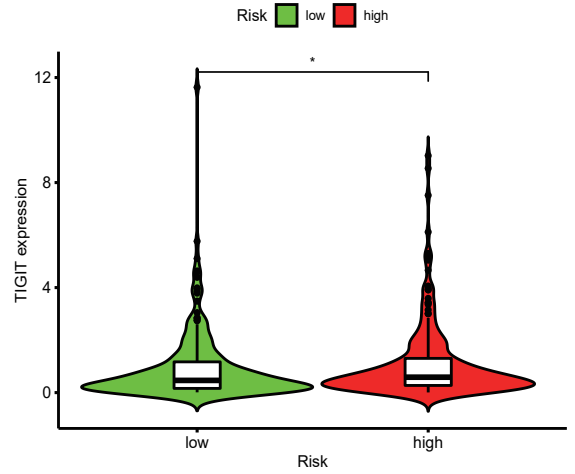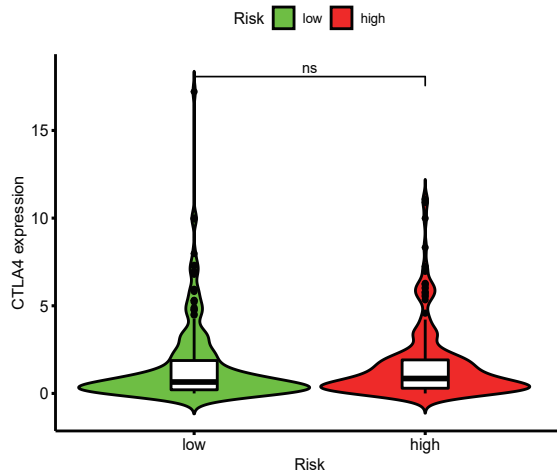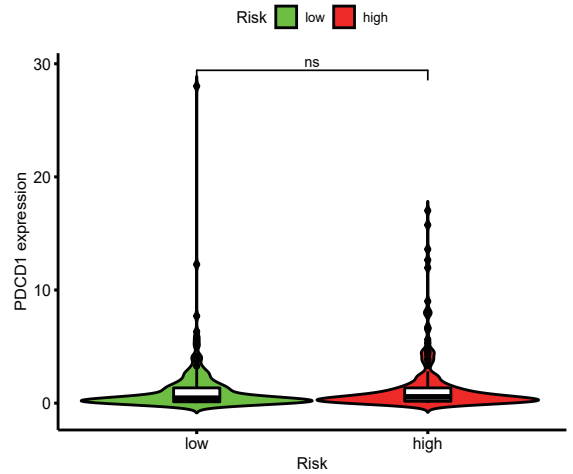

Supplement: Supplementary 5 — Supplementary Figure 5: the expression of key immune checkpoint genes between high- and low-risk groups. [file 7056506.f5.pdf]

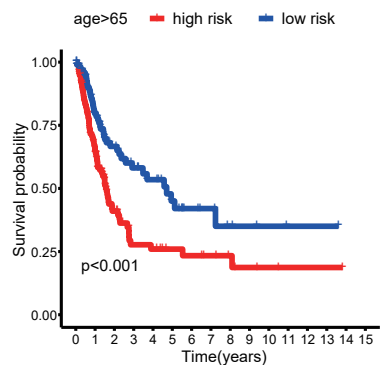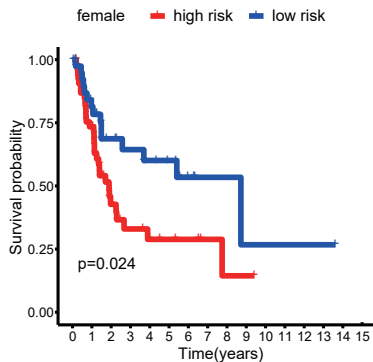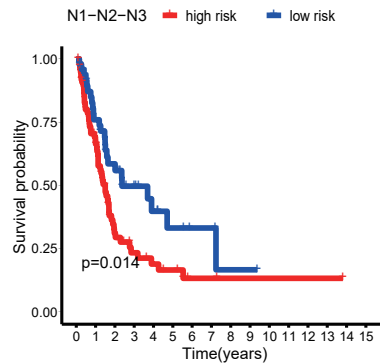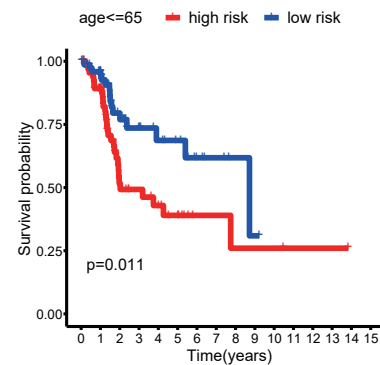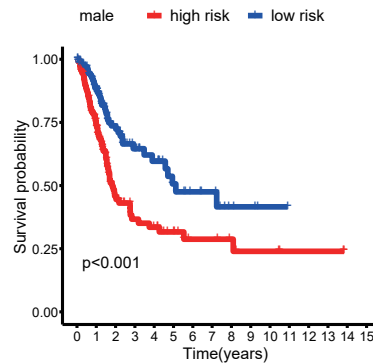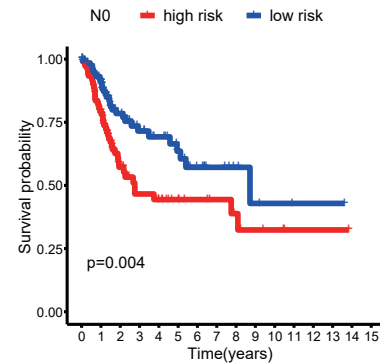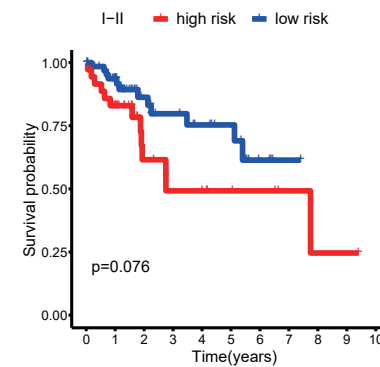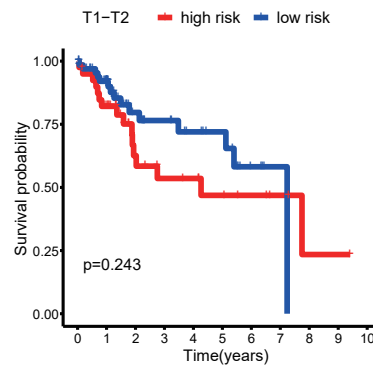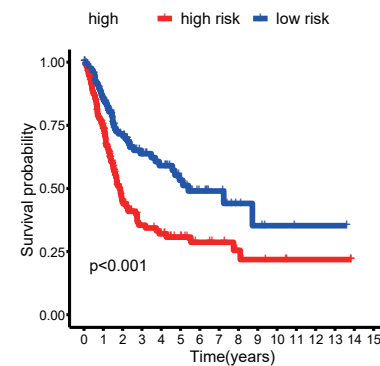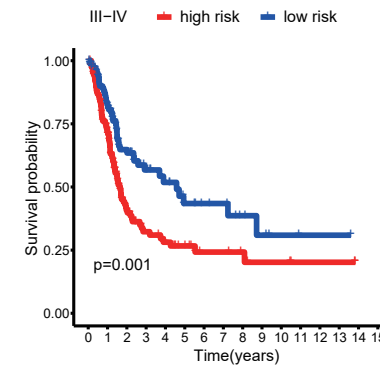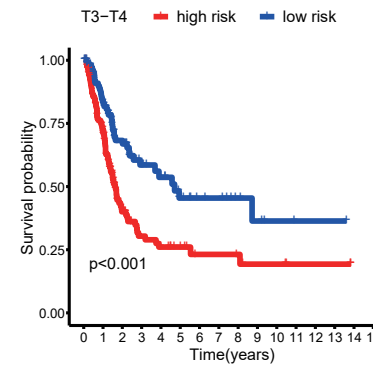

Supplement: Supplementary 6 — Supplementary Figure 6: Kaplan-Meier curve analyses showed the overall survival stratified by age, gender, TNM stage, grade, N stage, and T stage between the high-risk and low-risk groups. [file 7056506.f6.pdf]
